# Single-neuron diversity generated by *Protocadherin-β* cluster in mouse central and peripheral nervous systems

**DOI:** 10.3389/fnmol.2012.00090

**Published:** 2012-08-31

**Authors:** Keizo Hirano, Ryosuke Kaneko, Takeshi Izawa, Masahumi Kawaguchi, Takashi Kitsukawa, Takeshi Yagi

**Affiliations:** ^1^KOKORO-Biology Group, Laboratories for Integrated Biology, Graduate School of Frontier Biosciences, Osaka University, 1-3 Yamadaoka, SuitaOsaka 565-0871, Japan; ^2^Institute of Experimental Animal Research, Gunma University Graduate School of Medicine, 3-39-22 Showa-machi, MaebashiGunma 371-8511, Japan; ^3^Department of Ultrastructural Research, National Institute of Neuroscience, KodairaTokyo 187-8551, Japan; ^4^CREST, Osaka University, 1-3 Yamadaoka, SuitaOsaka 565-0871, Japan

**Keywords:** single-neuron diversity, neuronal individuality, protocadherin, Pcdh, monoallelic, combinatorial expression, single-cell 3′-RACE, neural circuit

## Abstract

The generation of complex neural circuits depends on the correct wiring of neurons with diverse individual characteristics. To understand the complexity of the nervous system, the molecular mechanisms for specifying the identity and diversity of individual neurons must be elucidated. The clustered *protocadherins* (*Pcdh*) in mammals consist of approximately 50 *Pcdh* genes (*Pcdh-α*, *Pcdh-β*, and *Pcdh-γ*) that encode cadherin-family cell surface adhesion proteins. Individual neurons express a random combination of *Pcdh-α* and *Pcdh-γ*, whereas the expression patterns for the *Pcdh-β* genes, 22 one-exon genes in mouse, are not fully understood. Here we show that the *Pcdh-β* genes are expressed in a 3′-polyadenylated form in mouse brain. *In situ* hybridization using a pan-*Pcdh-β* probe against a conserved *Pcdh-β* sequence showed widespread labeling in the brain, with prominent signals in the olfactory bulb, hippocampus, and cerebellum. *In situ* hybridization with specific probes for individual *Pcdh-β* genes showed their expression to be scattered in Purkinje cells from P10 to P150. The scattered expression patterns were confirmed by performing a newly developed single-cell 3′-RACE analysis of Purkinje cells, which clearly demonstrated that the *Pcdh-β* genes are expressed monoallelically and combinatorially in individual Purkinje cells. Scattered expression patterns of individual *Pcdh-β* genes were also observed in pyramidal neurons in the hippocampus and cerebral cortex, neurons in the trigeminal and dorsal root ganglion, GABAergic interneurons, and cholinergic neurons. Our results extend previous observations of diversity at the single-neuron level generated by *Pcdh* expression and suggest that the *Pcdh-β* cluster genes contribute to specifying the identity and diversity of individual neurons.

## Introduction

The central and peripheral nervous systems contain an enormous number of neurons, that have diverse characteristics and unique identities that enable them to generate complex neural circuits. Therefore, elucidating the molecular mechanisms that specify the unique characteristics of individual neurons is important for understanding the overall complexity of the nervous system.

Recent studies reported that two families of cell-cell recognition molecules, Dscam1 in insects and clustered protocadherin (*Pcdh*) in vertebrates, are promising candidates for specifying the unique characteristics of individual neurons (Zipursky and Sanes, 2010; Yagi, 2012). Dscam1 proteins are single-pass transmembrane domain proteins of the immunoglobulin superfamily that play important roles in neural circuit formation (Hattori et al., 2008; Schmucker and Chen, 2009). The fly *Dscam1* gene encodes 19,008 different ectodomain isoforms, which are expressed in a biased stochastic fashion in individual neurons, thereby conferring a unique molecular identity on each *Drosophila* neuron (Neves et al., 2004). In addition, Hattori et al. (2007) showed that *Dscam1* diversity is essential for generating complex neural circuits.

Approximately 100 of the diverse cadherin-superfamily genes are highly expressed in vertebrate brain (Takeichi, 2007; Yagi, 2008), and about 50 of these are encoded by the *Pcdh* gene clusters, *Pcdh-α*, *Pcdh-β*, and *Pcdh-γ* (Kohmura et al., 1998; Wu and Maniatis, 1999; Sugino et al., 2000; Hirayama and Yagi, 2006; Morishita and Yagi, 2007). Cadherin-superfamily proteins play key roles in the morphogenesis and function of the brain, including the formation and maintenance of the neuroepithelium, neurite extension, migration of neuronal cells, synaptogenesis, and synaptic plasticity (Takeichi, 2007; Suzuki and Takeichi, 2008). Previous studies revealed that the clustered *Pcdh*s play important roles in neuronal survival, axonal projection, synaptic connectivity, and several brain functions, including learning and memory (Wang et al., 2002b; Fukuda et al., 2008; Hasegawa et al., 2008; Katori et al., 2009). Since the clustered *Pcdh*s show unique gene regulations and form heteromultimeric Pcdh protein tetramers with homophilic binding specificity, the clustered *Pcdh*s are considered to be candidates for generating complex neural circuitry by providing molecular codes that determine neuronal individuality in the vertebrate central and peripheral nervous systems (Schreiner and Weiner, 2010; Zipursky and Sanes, 2010; Yagi, 2012).

The *Pcdh-α*, *Pcdh-β*, and *Pcdh-γ* gene clusters are tandemly localted on the mouse 18 chromosome. All isoforms of *Pcdh-α*, *Pcdh-β*, and *Pcdh-γ* genes possess their own promoters and variable exons in each gene cluster in the same direction (Wu et al., 2001). The *Pcdh-α* or *Pcdh-γ* variable exons are spliced onto each *Pcdh-α* or *Pcdh-γ* constant exons, respectively, that encode the common 3′ regions. In contrast, the *Pcdh-β* gene cluster does not contain constant exons and is instead comprised of unspliced single exon genes.

Extensive investigation of the gene regulation mechanisms in the clustered *Pcdh*s has identified some unique features. *In situ* hybridization analysis showed that the *Pcdh-α* and *Pcdh-γ* genes are differentially regulated at the level of individual neurons (Kohmura et al., 1998; Esumi et al., 2005; Frank et al., 2005), and single-cell analysis of Purkinje cells using multiplex RT-PCRs established that one or two variable exons in the *Pcdh-α* and *Pcdh-γ* clusters are stochastically expressed from each allele in individual Purkinje cells (Esumi et al., 2005; Kaneko et al., 2006). The expression of the clustered *Pcdh* genes is governed by the activation of individual promoters from among multiple promoters in the cluster (Tasic et al., 2002; Wang et al., 2002a). Furthermore, the discovery of a long-range regulatory element in the *Pcdh-α* (Ribich et al., 2006; Kehayova et al., 2011) and *Pcdh-β* (Yokota et al., 2011) gene clusters provided further support for a model in which promoter choice among multiple promoters drives unique expression patterns in single neurons.

A number of transcription factors or DNA-binding proteins have been implicated in the regulation of clustered *Pcdh* gene transcription: that is, *CTCF* (Golan-Mashiach et al., 2012; Hirayama et al., 2012; Monahan et al., 2012), *NRSF/REST* (Tan et al., 2010; Kehayova et al., 2011), *SOX4* (Castillo et al., 2012), *Mecp2* (Chahrour et al., 2008; Miyake et al., 2011), *Egr-1* (Schippert et al., 2009), and *Nipbl* (Kawauchi et al., 2009). In addition, *Pcdh* promoter CpG methylation is inversely correlated with *Pcdh* expression (Tasic et al., 2002; Kawaguchi et al., 2008; Dallosso et al., 2009, 2012; Kaneko et al., 2009). These results are consistent with the idea that the *Pcdh* cluster genes and their sophisticated gene regulation mechanisms could be involved in establishing the unique molecular identities of individual neurons in the brain (Chess, 2005; Zipursky and Sanes, 2010; Yagi, 2012). Although the regulatory mechanisms for generating *Pcdh* diversity at the single-neuron level have been addressed to some extent, whether this diversity contributes to the functional complexity of the mammalian nervous system remains to be determined.

To date, little information has been published on the expression of the *Pcdh-β* genes, which consist of 22 single exon genes located on mouse chromosome 18 (Vanhalst et al., 2001), although the postsynaptic localization of one Pcdh-β protein, Pcdh-β16, was reported for mouse and primate retina (Junghans et al., 2008; Puller and Haverkamp, 2011). Here, we show that the expression patterns of the *Pcdh-β* genes are similar to those of the *Pcdh-α* and *Pcdh-γ* genes. 3′-RACE analysis of single Purkinje cells showed that *Pcdh-β* isoforms derived from monoallelic chromosomes were combinatorially expressed in individual Purkinje cells. Furthermore, *in situ* hybridization analysis showed, for the first time, scattered expression patterns at the level of individual neurons in the trigeminal ganglion, dorsal root ganglion, GABAergic interneurons, and cholinergic neurons. Our results extend previous findings on the single-neuron diversity generated by *Pcdh* expression and suggest that *Pcdh-β* cluster genes may contribute to specifying the identity and diversity of individual neurons.

## Materials and methods

### Animals

C57BL/6 (B6) mice were purchased from Charles River Japan. F1 hybrid offspring were obtained by intercrossing mice of the laboratory strain B6 with the Japanese wild mouse strain JF1, obtained from the National Institute for Genetics (Mishima, Shizuoka, Japan). All procedures undertaken in this study were approved by our institute's Animal Care and Use Committee and conform to Japanese guidelines.

### RT-PCR

Total RNA was prepared from P21 B6 mouse cerebellum using Trizol reagent (Invitrogen) and reverse-transcribed by Superscript III reverse transcriptase (Invitrogen), using random primers, according to the manufacturer's protocol. The PCR of each *Pcdh-β* isoform was performed using cDNA as the template, 2 μl of 10× Ex-taq PCR buffer, 2 μl of 2.5 mM each dNTP mix, 0.4 μl of RT-PCR primer set, 0.1 μl of Ex-taq HS polymerase (Takara, Japan), and water to a final volume of 20 μl. The PCR conditions were 3 min at 94°C and then 35 cycles of 30 s at 94°C, 30 s at 60°C (for *Pcdh-β1*) or 58°C (for other *Pcdh-β* genes), and 2 min at 72°C. The primer sets and sequences are shown in Table S1.

### *In situ* hybridization

*In situ* hybridization was performed as described previously (Esumi et al., 2005; Kaneko et al., 2006; Watakabe et al., 2007; Katori et al., 2009; Noguchi et al., 2009; Watakabe et al., 2010; Yokota et al., 2011) on 10-μm-thick frozen sagittal and coronal sections prepared from the B6 mouse using *Pcdh-β* highly conserved and isoform-specific cRNA probes. The mouse ages and regions examined are indicated in the figure legends. A probe for a highly conserved region of the *Pcdh-β2*–*β22* genes, pan-*Pcdh-β* probe, was designed based on the sequence in mouse β12 (nucleotides 1422–2152 according to GeneBank™ accession number NM_053137). The *Pcdh-β* isoform-specific probes were designed based on mouse *β3* (nucleotides 237–1364; GeneBank™ accession number NM_053128), *β15* (nucleotides 161–1362; GeneBank™ accession number NM_053140), *β16* (nucleotides 277–1293; GeneBank™ accession number NM_053141), *β19* (nucleotides 227–1147; GeneBank™ accession number NM_053144), and *β22* (nucleotides 213–1146; GeneBank™ accession number NM_053147). The similarity of each *Pcdh-β* isoform-specific probe to other *Pcdh-α*, *Pcdh-β*, and *Pcdh-γ* genes and to other mouse genes was low enough (less than 65%) to detect specific *Pcdh-β* transcripts of interest. These specific isoform sequences were amplified using the primers shown in Supplemental Table S1 (for *in situ* hybridization probe) and KOD-plus polymerase (Toyobo, Japan). The PCR product was cloned into the *pCRII* vector (Invitrogen), and the sequence was confirmed by DNA sequencing. The resultant plasmids were used as templates to synthesize digoxigenin (DIG)-11-UTP-labeled (Roche) cRNA probes.

To obtain tissue samples, mice were deeply anesthetized with diethyl ether, then the brain was removed, embedded in O.C.T. compound (Sakura), and quickly frozen in isopentane cooled with dry ice. Sections (10-μm thick) were cut on a cryostat (Leica CM3050), thaw-mounted on slides (Matsunami), and air-dried. The sections were fixed in 4% paraformaldehyde (PFA) in 0.1 M phosphate buffer (PB, pH 7.3) for 10 min, washed three times in PBS, pH 7.4, acetylated for 10 min in 0.25% acetic anhydride in 0.1 M triethanolamine-HCl, pH 8.0, and washed three more times with PBS. Prehybridization was performed with hybridization buffer [50% formamide, 5 × SSC (20 × SSC is 3 M NaCl, 0.3 M sodium citrate, pH 7.0), 5× Denhardt's, 250 μg/ml yeast tRNA, 500 μg/ml salmon sperm DNA, and 0.2% RNasin RNase inhibitor (Promega)] for 30 min. The DIG-labeled cRNA probes were denatured for 5 min at 82°C and chilled on ice. The sections were covered with hybridization buffer containing 1 μg/ml of the cRNA probes, added drop wise, and a coverslip was added. The slides were incubated for 12 hrs at 72°C in a humidified chamber (50% formaldehyde, 5 × SSC), washed three times with 0.2 × SSC at 72°C, washed three times with TBS, pH 7.5 (100 mM Tris-HCl, pH 7.5, 100 mM NaCl), and rinsed with TNT (0.05% Tween 20 in TBS, pH 7.5). To detect the hybridized probes, the sections were blocked with 1× blocking solution (Roche) in TNT for 30 min, and then incubated with alkaline phosphatase (AP)-conjugated anti-DIG antibody (1:1000 dilution, Roche) in the blocking solution for 1 h. The sections were rinsed three times with TBS, pH 7.5, and the enzymatic activity was visualized with 0.2 mM 5-bromo-4-chloro-3-indolyl-phosphate, 0.2 mM nitro blue tetrazolium (NBT/BCIP) in 100 mM Tris-HCl, pH 9.5, 100 mM NaCl, 20 mM MgCl_2_, in the dark, until the signal reached a satisfactory intensity.

We also carried out double fluorescence *in situ* hybridization. Fluorescein (FITC)-labeled cRNA probes against glutamate decarboxylase 1 (*GAD67*), choline O-acetyltransferase (*ChAT*), and vesicular acetylcholine transporter (*VAChT*) were synthesized with a FITC-UTP RNA labeling Kit (Roche). The probes were designed based on mouse *GAD67* (nucleotides 1073 ~ 2012; GeneBank™ accession number NM_008077), rat *ChAT* (nucleotides 243 ~ 745; GeneBank™ accession number NM_001170593), and rat *VAChT* (*Slc18a3*) (nucleotides 1201 ~ 2194; GeneBank™ accession number NM_031663). The DIG-labeled *Pcdh-β* probe and a FITC-labeled *GAD67*, *ChAT*, or *VAChT* cRNA probe were hybridized to sections, which were washed as above.

To detect the FITC-labeled probes, the sections were incubated with an anti-FITC antibody conjugated with horseradish peroxidase (Jackson Immuno Research Laboratory, 1:4000 in the blocking buffer) for 2–5 hrs at room temperature. After being washed in TNT three times for 15 min, the sections were treated with 1:50 diluted TSA-Plus [dinitrophenol (DNP)] reagents for 5 min, according to the manufacturer's instructions (Perkin-Elmer, Wellesley, MA), which converted the FITC signals to DNP signals. After being washed in TNT three times for 10 min, the sections were incubated overnight at 4°C with an anti-DNP antibody conjugated with Alexa488 (1/500, Molecular Probes) in 1% blocking buffer. At this point, an anti-DIG antibody conjugated with AP (1:1000, Roche Diagnostics) was included in the incubation, to detect the DIG-labeled probes. The AP activity was visualized with NBT/BCIP (see above) or with HNPP fluorescence.

To detect the DIG-labeled probes with HNPP fluorescence, the sections were washed three times in TNT, once in TS 8.0 (0.1 M Tris-HCl, pH 8.0, 0.1 M NaCl, 50 mM MgCl_2_), and the AP activity was detected using an HNPP fluorescence detection set (Roche Diagnostics), according to the manufacturer's instructions. The incubation for this substrate was carried out for 30 min and stopped by washing in TS 8.0. The sections were then counterstained with 4′,6-diamidino-2-phenylindope, dihydrochloride (DAPI, Invitrogen, USA) diluted in TS 8.0–100 ng/ml for 3 min. After a brief wash in TS 8.0, the sections were mounted with CC/Mount (Diagnostic Biosystems, Pleasanton, CA) mounting medium.

### Split single-cell 3′-RACE of Purkinje cells

A split single-cell 3′-RACE method for single Purkinje cells was developed to analyze the one-exon *Pcdh-β3*, *β9*, *β10*, *β15*, *β19*, and *β22* genes, and was based on our previously published method for single-cell RT-PCR (Esumi et al., 2006). The primer sets and sequences are shown in Table S1 (for split single-cell 3′-RACE). In brief, Purkinje cells were prepared from the cerebellum of P21 mice, from the F1 litter of a B6 female × JF1 male. The tissue was dissected and digested with 90 units of papain (Worthington) at 37°C for 30 min in 10 ml of dissociation solution [0.002% DL-cysteine HCl (Sigma), 0.05% DNase I (Sigma), 0.1% bovine serum albumin (Sigma), and 0.05% glucose (Nacalai Tesque)]. The digested tissue was spun for 8 min at 300× g, and the pellet was resuspended in Dulbecco's modified Eagle's medium (Sigma). To remove debris, the cells were filtered through a 100-μm cell strainer (Falcon). Single Purkinje cells were picked up by glass capillary and placed in thin, 200-μl PCR tubes, with 6 μl of RNase-free water. Complementary DNA was synthesized from the single-cell samples after adding 12.5 pmol/reaction of adaptor-attached oligo dT primer and 2.5 pmol/reaction of *Pcp-2* RT primer in a total volume of 10 μl, using six units of Thermoscript reverse transcriptase (Invitrogen), according to the manufacturer's protocol, at 55°C for 60 min. The reaction was stopped by heating at 85°C for 5 min.

The cDNAs derived from a single Purkinje cell were split into three PCR tubes as 3.3-μl aliquots and used as a template for the first round of a multiplex PCR, which was performed using the 3.3 μl of cDNA, 2.5 μl of 10× Ex-taq PCR buffer, 2 μl of 2.5 mM each dNTP mix, 1.5 μl of the first PCR primer set, 0.1 μl of Ex-taq HS polymerase (Takara), and 15.6 μl of water. The PCR conditions were an initial 3 min at 95°C and then 28 cycles of 20 s at 95°C, 20 s at 58°C, and 5 min at 68°C. The first PCR products were split into 0.5-μl aliquots, which were used as the template in the second round of nested PCR with 2 μl of 10× Ex-taq PCR buffer, 1.6 μl of 2.5 mM each dNTP mix, 0.4 μl of the second PCR primer set, 0.1 μl of Ex-taq HS polymerase (Takara), and 15.4 μl of water. The PCR conditions for the *Pcdh-β* genes were an initial 3 min at 96°C, 40 cycles of 30 s at 96°C, 15 s at 56°C (for *β3*, *β9*, and *β15*) or 58°C (for *β10*, *β19*, and *β22*), and 1 min at 72°C. The PCR conditions for the *β-actin* and *Pcp-2* were described in previous reports (Esumi et al., 2005, 2006; Kaneko et al., 2006). The primer sets and sequences are shown in Table S1.

The secondary PCR products were divided into two tubes. One PCR-amplified product was analyzed by agarose gel electrophoresis: before loading, the sample was mixed with electrophoresis loading buffer (Takara) and heated to 72°C for 5 min, to sharpen the electrophoretic bands. The other half of the PCR sample was purified by polyethylene glycol precipitation and sequenced by direct sequencing, using a BigDye™ DNA sequencing kit (version 3.1) (ABI). The data were analyzed on an ABI Prism 3100 or an ABI Prism 3730 Genetic Analyzer. Direct sequencing permitted us to identify the allele from which each purified PCR product was derived. The discrimination of monoallelic expression from biallelic expression was accomplished using SeqScape software (ABI). The sequencing results from each 3′-RACE PCR product were confirmed by performing direct sequencing twice, using two distinct pairs of forward and reverse primers.

## Results

### Expression of the *Pcdh-β* transcripts in mouse brain

**Figure 1:**
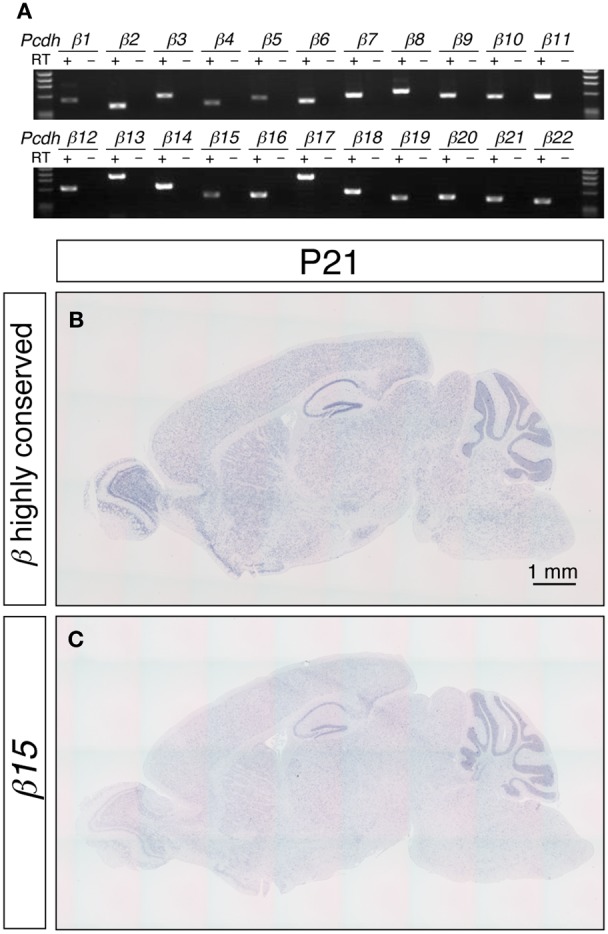
**Expression of the *Pcdh-β* transcripts in the mouse brain. (A)** RT-PCR analysis of P21 cerebellum using isoform-specific primers demonstrated that all 22 of the *Pcdh-β* transcripts were expressed. *In situ* hybridization analysis with a *Pcdh-β* highly conserved probe **(B)** and with a probe specific for *β15* **(C)**. Sagittal sections of P21 mouse brain are shown. The distributions of the signals were almost identical, but the intensity of the *β15*-specific probe at the macroscopic level was weaker than that obtained with the *Pcdh-β* highly conserved probe, suggesting that the expression of the *Pcdh-β* transcripts overlapped at the macroscopic level.

To examine the expression pattern of each *Pcdh-β* gene in the brain, we first carried out an RT-PCR analysis of all 22 *Pcdh-β* gene family exons, using cDNA from the P21 mouse cerebellum (Figure 1A). This analysis clearly showed that all 22 *Pcdh-β* gene family exons were transcribed in the cerebellum. Next, we identified the 3′ ends of some of the *Pcdh-β* transcripts by 3′-RACE analysis using RNA from P21 mouse cerebellum (Table S2). No spliced product of *Pcdh-β* with constant region exons of *Pcdh-α* or *Pcdh-γ* was detected by first-round RT-PCR analysis (data not shown). These data re-confirmed that the *Pcdh-β* genes are transcribed as 3′ polyadenylated mRNAs (Vanhalst et al., 2001).

The distribution of the *Pcdh-β* transcripts in the P21 mouse brain was examined by *in situ* hybridization (Figures 1B,C). Because the DNA sequence that corresponds to extracellular cadherin domain 4 (EC4)-EC5-EC6 of *Pcdh-β12* is highly conserved from *β2* to *β22* (more than 84% similarity), we used this sequence as a pan-*Pcdh-β* probe (*Pcdh-β* highly conserved probe, hereafter) to detect all 21 isoforms of *Pcdh-β*. We excluded the *β1* gene from this study because of its low similarity to *β2*–*β22*. The *Pcdh-β* highly conserved probe hybridized widely in the brain, and prominent signals were observed in the olfactory bulb, hippocampus, and cerebellum. No signal was observed with the sense probe (data not shown). These results are consistent with findings obtained for rat brain (Sago et al., 1995).

To examine the distribution of each member of the *Pcdh-β* subfamily genes, we performed an *in situ* hybridization analysis using probes that detected specific *Pcdh-β* isoforms. Sequences showing low similarity (<65%) with other *Pcdh-β* gene members were used as probes. Although the *Pcdh-β* genes are very similar to each other, the similarity of *β3*, *β15*, *β16*, *β19*, and *β22* is relatively low, and could be reliably discriminated. When examined at low magnification, the signals for *β15* (Figure 1C) and the other 4 *Pcdh-β* genes (data not shown) hybridized in a pattern that was identical to that obtained using the *Pcdh-β* highly conserved probe, but with lower signal intensity. These results suggested that the *Pcdh-β* isoforms, at least *β3*, *β15*, *β16*, *β19*, and *β22*, have macroscopically overlapping expression patterns in the mouse brain.

### Monoallelic and combinatorial gene regulation of *Pcdh-β* genes in single Purkinje cells

**Figure 2:**
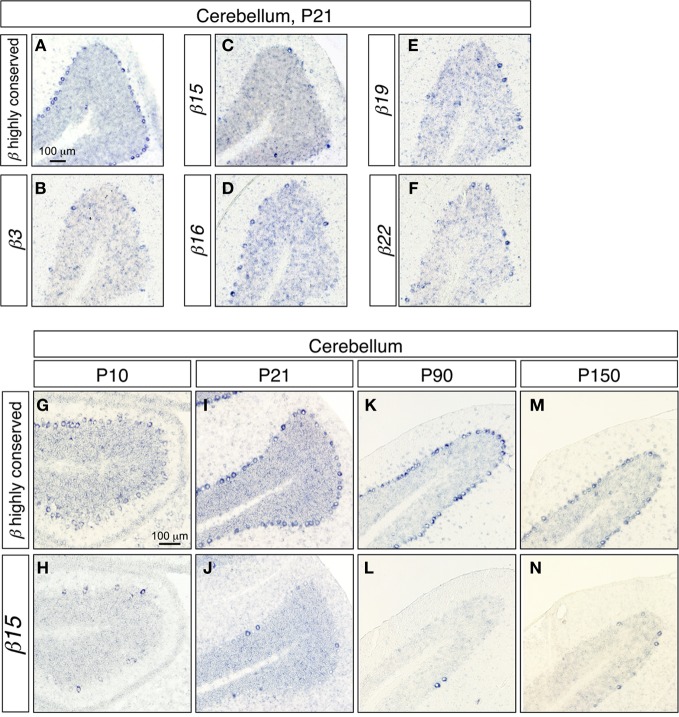
***In situ* hybridization analysis of *Pcdh-β* in the mouse cerebellum. (A–F)** Coronal sections. **(A)** *In situ* hybridization with the *Pcdh-β* highly conserved probe; **(B–F)** *in situ* hybridization using probes specific for *β3* **(B)**, *β15* **(C)**, *β16* **(D)**, *β19* **(E)**, and *β22* **(F)**. **(G–M)** Comparison of staining with the *Pcdh-β* highly conserved probe **(G,I,K,M)** and the *β15*-specific probe **(H,J,L,N)** at P10–P150.

To investigate the gene regulation of the *Pcdh-β* cluster, we examined the expression patterns of the *Pcdh-β* transcripts in cerebellar Purkinje cells, since the regulation of the *Pcdh-α*, and *Pcdh-γ* clusters was extensively studied in these cells (Esumi et al., 2005; Kaneko et al., 2006). In the P21 cerebellum, the *Pcdh-β* highly conserved probe intensely stained most Purkinje cells (Figure 2A). In contrast, the specific probes for *β3*, *β15*, *β16*, *β19*, and *β22* stained only a subset of the Purkinje cells (Figures 2B–F), showing that the *Pcdh-β* isoforms, at least *β3*, *β15*, *β16*, *β19*, and *β22*, were expressed in a scattered pattern among the Purkinje cells. Next, we analyzed the age-dependence of the expression patterns of the *Pcdh-β* genes in Purkinje cells. From P10 to P150, all the Purkinje cells were intensely stained by the *Pcdh-β* highly conserved probe (Figures 2A,G,I,K,M), whereas the specific probe for *β15* stained only a subset of Purkinje cells (Figures 2C,H,J,L,N). Likewise, the specific probes for *β3*, *β16*, *β19*, and *β22* stained only subsets of the Purkinje cells, resulting in scattered patterns such as those seen at P21 (data not shown). Therefore, the expression of the *β3*, *β15*, *β16*, *β19*, and *β22* isoforms, and probably all of the *Pcdh-β* isoforms, was scattered among Purkinje cells from P10 to P150. On the other hand, in granule cells stained signals for both *Pcdh-β* highly conserved and specific *β15* probes were reduced in P90 and P150.

The next step was to confirm the differential expression revealed by the *in situ* hybridization, and to further investigate whether the gene regulation of *Pcdh-β* transcripts in single neurons is monoallelic and combinatorial. For this purpose, we modified our split single-cell RT-PCR method. Since the *Pcdh-β* genes consist of only one exon (Vanhalst et al., 2001), conventional RT-PCR against *Pcdh-β* exon could amplify not only the *Pcdh-β* transcripts but also the genomic DNA, leading us to erroneous conclusions. Fortunately, the *Pcdh-β* transcripts are polyadenylated (Table S2), so we adapted our method to 3′-RACE. First, we searched for a region of strong similarity shared by several of the *Pcdh-β* genes to use in the first multiplex primer annealing and found that base pairs 2289–2307 of *β15* are highly conserved in *β3*, *β9*, *β10*, *β15*, *β19*, and *β22*. We next sequenced the region proximal to the functional polyadenylation signal of these six *Pcdh-β* genes in the JF1 mouse strain, and detected polymorphisms against the B6 strain (Figure S1). These data ultimately led us to choose *β3*, *β9*, *β10*, *β15*, *β19*, and *β22* as the targets for this analysis.

We tested our split single-cell 3′-RACE method using Purkinje cells of P21 F1 mice from a B6 × JF1 cross. After optimizing the experimental conditions, we reproducibly obtained the predicted fragments for *β3*, *β9*, *β10*, *β15*, *β19*, *β22*, *β-actin* (positive control for 3′-RACE amplification), and *Pcp-2* (a marker for Purkinje cells). Only single Purkinje cell-derived cDNA samples that showed intense expressions of *Pcp-2* and *β-actin* were used for the expression analysis of the individual *Pcdh-β* isoforms in subsequent experiments.

**Figure 3:**
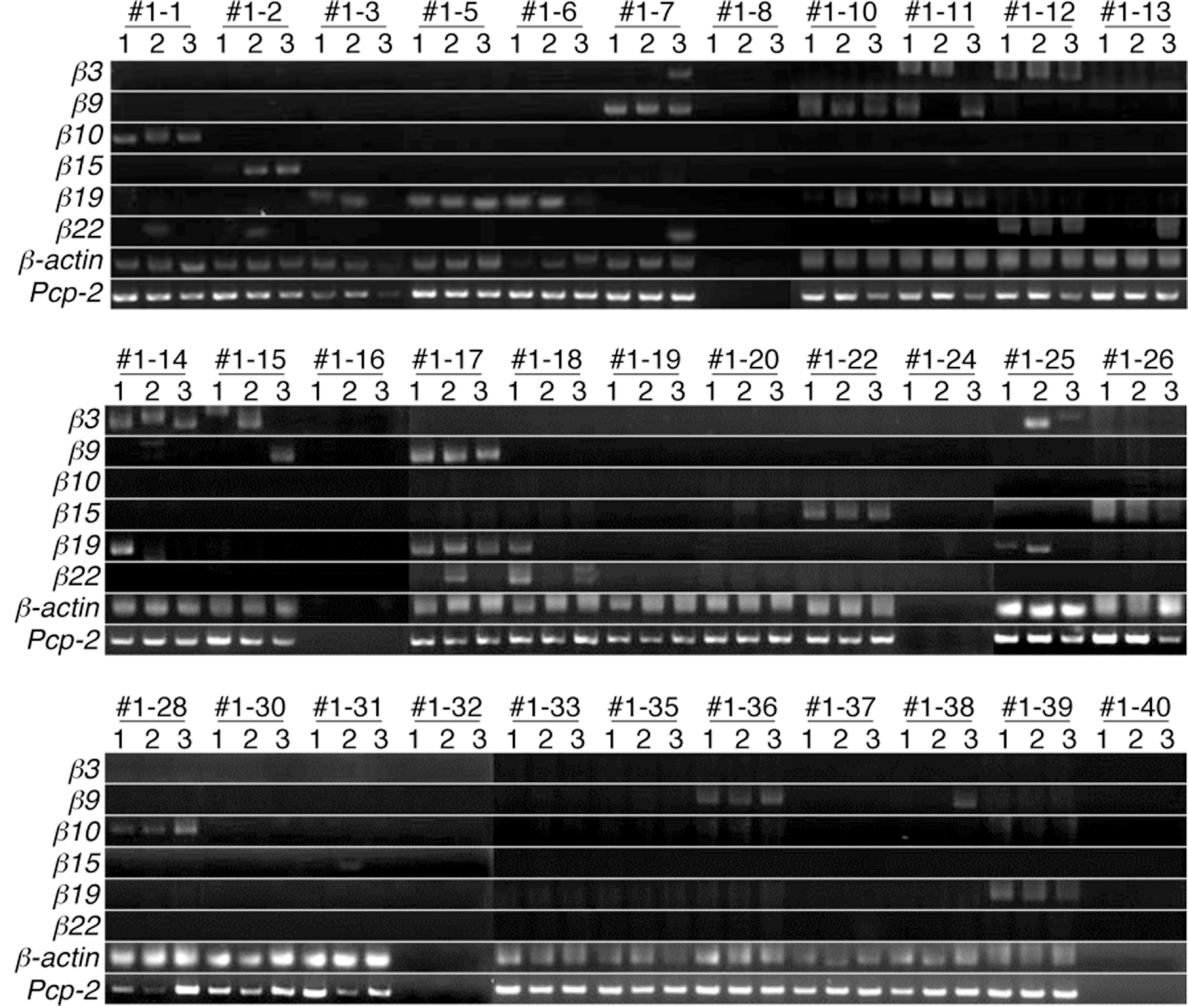
**Split single-cell 3′-RACE analysis of *Pcdh-β* in Purkinje cells.** Purkinje cells were from the P21 F1 progeny of a cross between the B6 and JF1 strains. Individual Purkinje cells are indicated as #1–1 to #1–39. Samples #1–8, #1–16, #1–24, #1–32, and #1–40 were negative controls that contained no cells. The RNAs in each Purkinje cell were reverse transcribed using an adapter-attached oligo dT primer and a *Pcp-2*-specific primer set. Each RT sample was divided into three separate tubes and subjected to 3′-RACE (PCR). The three independent PCR products are referred to as 1, 2, and 3. The results for electrophoresis of the second-round 3′-RACE (PCR)-products are shown.

Next, we analyzed the expression patterns of the *β3*, *β9*, *β10*, *β15*, *β19*, and *β22* transcripts in single Purkinje cells using the split single-cell 3′-RACE method (Figure 3). Of the 28 single Purkinje cells analyzed, all were positive for the PCR products of *Pcp-2* and *β-actin*, confirming that they were mature Purkinje cells, and their mRNAs were successfully reverse-transcribed. None of the control samples (#1–8, #1–16, #1–24, #1–32, or #1–40), which did not contain Purkinje cells, yielded PCR products, confirming the reliability of this method.

When the cells were subjected to the *Pcdh-β* gene-specific second-round PCR, 21 cells were positive for some of the *Pcdh-β* genes. Of these, 15 showed specific amplification of one of the six *Pcdh-β* genes investigated in all three (3/3) sample tubes (for example, *β10* in Cell #1–1, *β15* in Cell #1–2, and *β19* in Cell #1–5). In addition to the 3/3 PCR amplifications, five Purkinje cells showed specific PCR amplifications in 2/3 tubes (for example, *β19* in Cells #1–3, *β3* in Cell #1–15, and *β22* in Cell #1–18), and specific PCR amplification was seen in 1/3 tubes for two Purkinje cells (*β22* in Cell #1–13 and *β9* in Cell #1–38). Obtaining only 2/3 and 1/3 positive tubes may suggest that these cells contained a low amount of the corresponding mRNAs. The remaining seven cells were negative for any of the *Pcdh-β* genes, probably indicating that other *Pcdh-β* genes not included in this analysis were expressed by these cells. Finally, more than one *Pcdh-β* isoform was amplified in 3/3 tubes of three Purkinje cells (*β9* and *β19* in Cell #1–10, *β3* and *β22* in Cell #1–12, and *β9* and *β19* in Cell #1–17). These results strongly suggested that the *Pcdh-β* genes are expressed in Purkinje cells in a differential and combinatorial manner.

**Figure 4:**
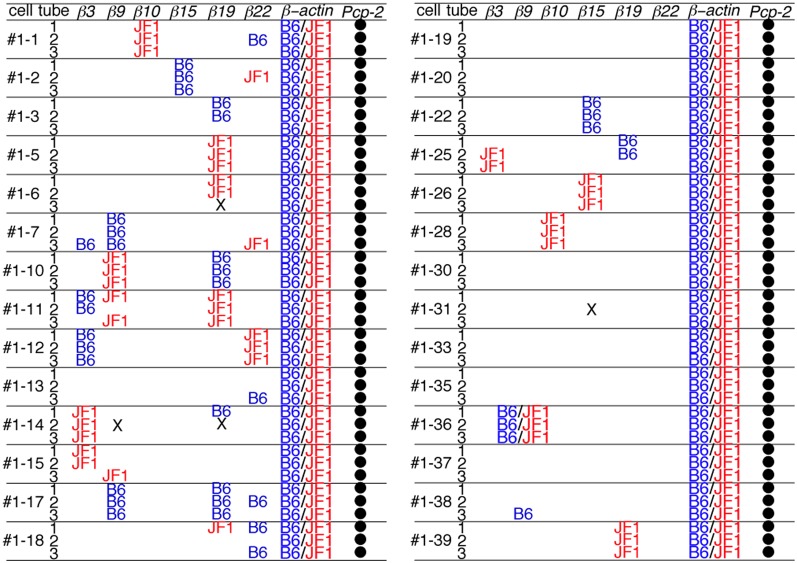
**Distribution of the B6 and JF1 alleles as the source of each *Pcdh-β* gene in individual Purkinje cells.** To determine whether the amplified 3′-RACE (PCR) products were from the B6 or JF1 allele, we performed direct sequencing of the products in each of the three tubes from 28 Purkinje cells. The samples that contained an insufficient quantity of product for sequencing are indicated with an X. The amplified *Pcp-2* products were not sequenced and are indicated with a closed circle.

Next, each PCR product was directly sequenced to distinguish among three possible patterns of expression: monoallelic expression of the maternal allele, monoallelic expression of the paternal allele, and biallelic expression. In the case of *β-actin*, all the PCR products showed a biallelic expression pattern. This was consistent with our previous analysis (Esumi et al., 2005; Kaneko et al., 2006), which was obtained by split single-cell RT-PCR. Thus, these findings confirmed the reliability of the present method. In contrast to *β-actin*, a monoallelic expression pattern was found for the *Pcdh-β* genes in 17 of the 18 3/3 3′-RACE-positive products (94%) (Figure 4) as follows: *β10* in Cell #1–1, *β15* in Cell #1–2, *β19* in Cell #1–5, *β9* in Cell #1–7, *β19* in Cell #1–5, *β9* and *β19* in Cell #1–10, *β19* in Cell #1–11, *β3* and *β22* in Cell #1–12, *β3* in Cell #1–14, *β9* and *β19* in Cell #1–17, *β15* in Cell #1–22, *β15* in Cell #1–26, *β10* in Cell #1–28, *β19* in Cell #1–39. Biallelic expression was observed only for *β9* in Cell #1–36. Statistically, if this assay amplified only a single cDNA molecule, even though biallelic products were present in the PCR tube, an artifactual 3/3 monoallelic expression pattern would theoretically be observed for 25% of the cells (Rhoades et al., 2000). Clearly, at 94% vs. 25%, there was a highly significant difference, which was upheld by a χ–square analysis between monoallelic and biallelic expression (*P* < 10^−4^). Of the 17 monoallelically expressed genes, seven were derived from the B6 allele and 10 from the JF1 allele. Thus, no allelic distortion from the paternal or maternal allele was found in this assay. Taken together, these results clearly showed that the *Pcdh-β* cluster genes are regulated monoallelically and combinatorially in individual Purkinje cells. This gene regulation would provide the molecular basis for the random expression of the *Pcdh-β* cluster genes, similar to the *Pcdh-α* and *Pcdh-γ* clusters.

### Diversity of the 3′-terminus of the *Pcdh-β* transcripts within single Purkinje cells

**Figure 5:**
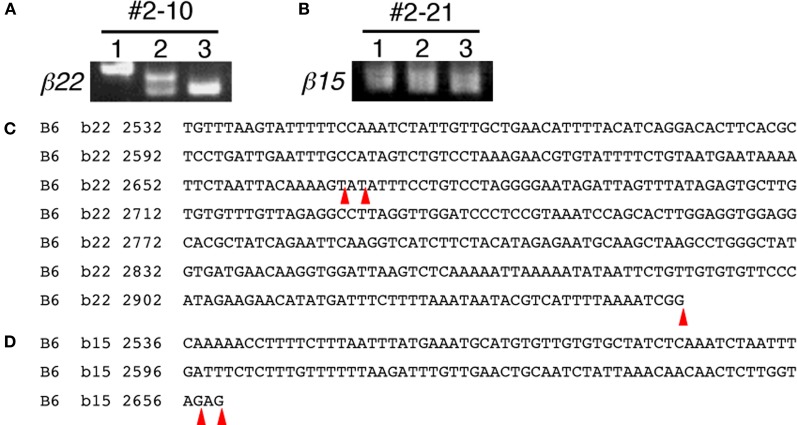
**3′-terminal diversity of *Pcdh-β* transcripts within single Purkinje cells.** Single-cell 3′-RACE analysis of *β22* in Cell #2–10 (Figures 5A,C) and *β15* in Cell #2–21 (Figures 5B,D). The position of polyadenylation is indicated by red arrowheads. The *β22* transcripts in Cell #2–10 showed three types of 3′-termini. The *β15* transcripts in Cell #2–21 showed two types of 3′-termini.

During the expressional analysis of the *Pcdh-β* transcripts using single-cell 3′-RACE, we simultaneously analyzed whether the 3′-terminus of each *Pcdh-β* transcript in single neurons was homogeneous or heterogeneous (Figure 5). In most individual Purkinje cells, the 3′-terminus of each *Pcdh-β* transcript was homogeneous, although we observed 3′-terminal heterogeneity in two cells: *Pcdh-β22* in Cell #2–10 (Figures 5A,C) and *Pcdh-β15* in Cell #2–21 (Figures 5B,D). The *β22* transcripts in Cell #2–10 showed three types of 3′-termini, while the *β15* transcripts in Cell #2–21 showed two types of 3′-termini. These data indicate that the polyadenylation of each *Pcdh-β* gene within a single neuron is not always homogeneous, suggesting the existence of transcriptional diversity for each *Pcdh-β* gene.

### Scattered expression of *Pcdh-β* transcripts in the central and peripheral nervous systems

**Figure 6:**
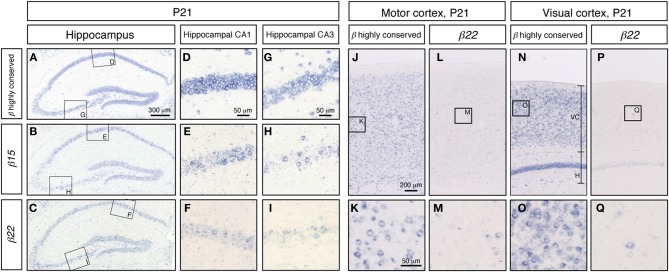
***In situ* hybridization analysis of *Pcdh-β* in mouse hippocampus and cerebral cortex.** *Pcdh-β* gene expression in P21 mouse hippocampus at low **(A,B,C)** and high **(D–I)** magnification, showing the CA1 and CA3 fields. **(A,D,G)** *β2* to *β22* expression using the *Pcdh-β* highly conserved probe. **(B,E,H)** *β15* expression. **(C,F,I)** *β22* expression. Examination of the cerebral motor cortex **(J–M)**, and cerebral visual cortex **(N–Q)** with specific probes for *β22* **(L,M,P,Q)**, and the *Pcdh-β* highly conserved probe **(J,K,N,O)**. Most neurons were stained by the *Pcdh-β* highly conserved probe, but the specific probes stained only subsets of these neurons, suggesting that each member of the *Pcdh-β* subfamily is expressed differentially in subpopulations of these neurons.

To extend our knowledge of the neuron types that showed random expression of the *Pcdh-β* cluster genes, which might correspond to the specification of individual neuron identity, we examined the expression of *Pcdh-β* genes in a wide range of neurons using *in situ* hybridization. In the P21 hippocampus, intense signals from the *Pcdh-β* highly conserved probe were observed in all the pyramidal cell–layer neurons in CA1 and CA3 (Figures 6A,D,G). In contrast, the specific probes for *β15* and *β22* stained only subsets of these neurons (Figures 6B,E,H, and C,F,I, respectively). The other specific probes (for *β3*, *β16*, and *β19*) also stained only subsets of these neurons (data not shown). Furthermore, in the P21 cerebral cortex, most neurons showed intense staining for the *Pcdh-β* highly conserved probe (see Figures 6J and K for staining in the P21 motor cortex and Figures 6N and O for staining in the P21 visual cortex). In contrast, the specific probe for *β22* stained only a subset of neurons in each of these regions (Figures 6L,M,P,Q), as did the other specific probes (data not shown). These results clearly demonstrated that these *Pcdh-β* gene isoforms were expressed only in subsets of neurons in the cerebral cortex and of pyramidal neurons in the hippocampal CA1 and CA3 areas, indicating that these *Pcdh-β* gene isoforms are expressed randomly and differentially in these neurons.

**Figure 7:**
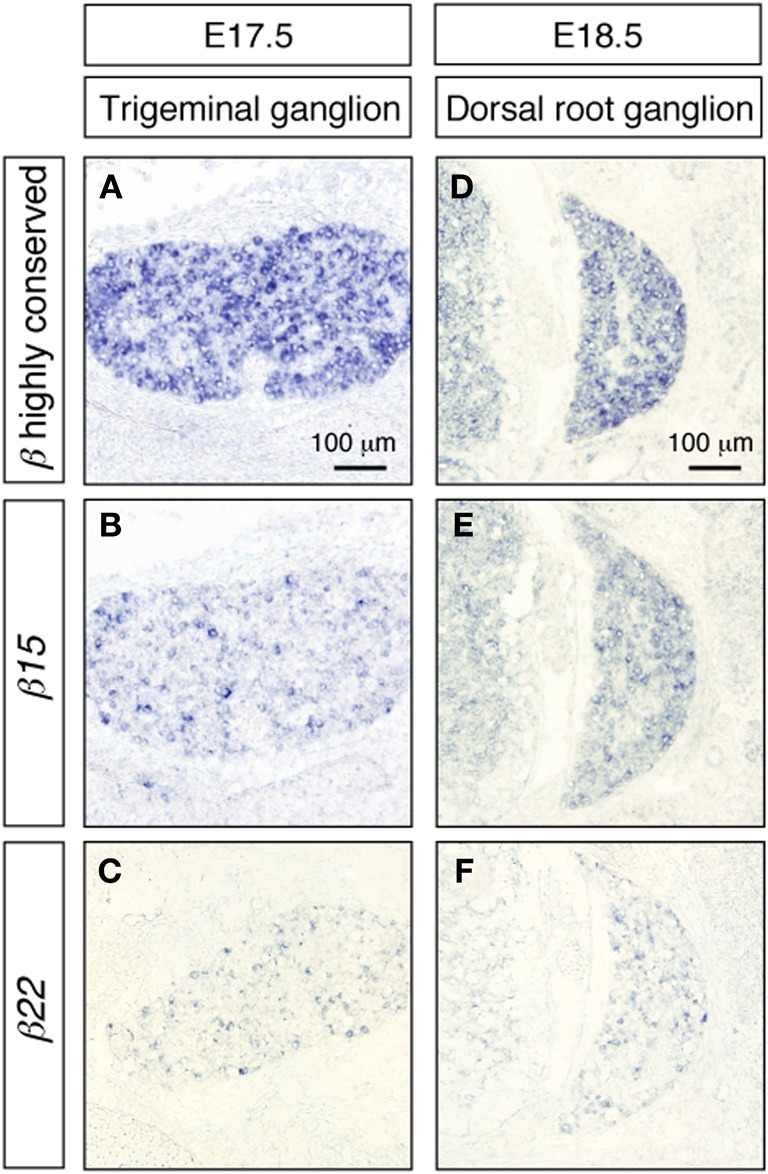
***In situ* hybridization analysis of *Pcdh-β* genes in mouse trigeminal and dorsal root ganglia.** *β2* to *β22* expression using the *Pcdh-β* highly conserved probe on embryonic mouse trigeminal ganglion **(A)** and dorsal root ganglion **(D)**. **(B,E)** Specific expression of *β15*; **(C,F)** specific expression of *β22*. Most neurons were stained by the *Pcdh-β* highly conserved probe, but the specific probes stained only subsets of these neurons, suggesting that each member of the *Pcdh-β* subfamily is expressed differentially in subpopulations of these neurons.

We also examined the expression of the *Pcdh-β* transcripts in the trigeminal and dorsal root ganglia. As in the cerebral cortex and the hippocampus, these peripheral nervous system neurons showed prominent signals from the *Pcdh-β* highly conserved probe (Figures 7A,D), while distinct subsets were labeled with the *β15* and *β22* probes (Figures 7B,C,E,F) as well as the *β3*, *β16*, and *β19* probes (data not shown). These results indicated that each *Pcdh-β* gene isoform is expressed randomly and differentially in the peripheral nervous system (at least in the trigeminal and dorsal root ganglia).

**Figure 8:**
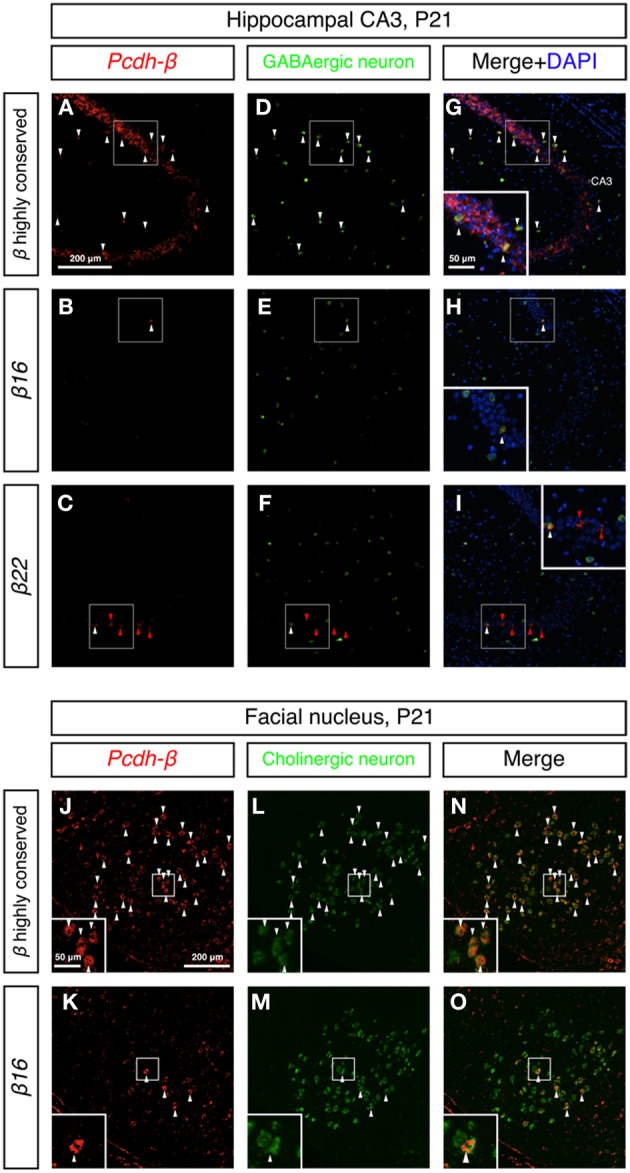
**Differential expression of *Pcdh-β* genes in GABAergic interneurons and cholinergic neurons. (A–I)** Double *in situ* hybridization analysis of *Pcdh-β* genes (red: HNPP fluorescence) and *GAD67* (green) in sagittal sections of P21 mouse hippocampus. **(J–O)** Double *in situ* hybridization analysis of *Pcdh-β* genes (red: false color of NBT/BCIP) and cholinergic neuron markers (*ChAT* + *VAChT*, green) in coronal sections of P21 mouse brain stem facial nucleus. Insets: High-magnification photomicrograph of the outlined area. Arrowheads indicate neurons expressing both *Pcdh-β* and *GAD67* or *ChAT* + *VAChT* mRNAs (white or black), and *β22* mRNA alone (red), respectively. Most neurons were stained by the *Pcdh-β* highly conserved probe, but the specific probes stained only subsets of these neurons, suggesting that each member of the *Pcdh-β* subfamily is expressed differentially in subpopulations of GABAergic interneurons in the hippocampus and cholinergic neurons in the facial nucleus.

To examine whether the *Pcdh-β* cluster genes showed random-scattered expression in GABAergic and cholinergic neurons, we performed double *in situ* hybridization analysis of the *Pcdh-β* genes with marker genes for GABAergic and cholinergic neurons. In the hippocampus, GABAergic interneurons constitute an inhibitory network that regulates excitatory neurons (Kullmann, 2011). The GABAergic interneurons were labeled with a *GAD67* probe (Figures 8D–F) and showed strong labeling with the *Pcdh-β* highly conserved probe (Figures 8A,G). Distinct subsets of GABAergic interneurons were labeled with the *β16* and *β22* probes (Figures 8B,H,C,I). In the brain stem facial nucleus, cholinergic neurons, which were labeled with *ChAT* + *VAChT* probes (Figures 8L,M) (Arvidsson et al., 1997), showed prominent labeling by the *Pcdh-β* highly conserved probe (Figures 8J,N), and distinct subsets were labeled with the *β16* probe (Figures 8K,O). Taken together, these results revealed that each member of the *Pcdh-β* cluster genes is expressed in a scattered pattern in a wide range of neuron types in the central and peripheral nervous systems in mouse.

## Discussion

In this study, we extended previous analyses on how *Pcdh* genes contribute to single-neuron diversity, by examining the expression patterns of the *Pcdh-β* genes using *in situ* hybridization analysis and the newly developed single-cell 3′-RACE analysis. Our results revealed that the *Pcdh-β* isoforms showed random-like scattered expression in both the central and the peripheral nervous systems, and that the *Pcdh-β* genes were regulated monoallelically and combinatorially in single Purkinje cells. We argue that the *Pcdh-α*, *Pcdh-β*, and *Pcdh-γ* gene clusters confer unique molecular identities on individual neurons that could be responsible for appropriate neuronal circuit formation.

The newly developed split single-cell 3′-RACE method revealed that a variety of 3′-ends of *Pcdh-β* mRNAs could be expressed by a single Purkinje cell. There are numerous examples of genes with multiple polyadenylation signals, and the 3′ ends of their transcripts sometimes vary with the developmental stage or state of the tissue in which they are expressed (Edwalds-Gilbert et al., 1997). However, it is unclear if the variation in the 3′-ends of mRNAs is constant or changeable within a single cell during a single time-frame. The present study revealed that some of the *Pcdh-β* genes have more than two functional polyadenylation signals, and that the polyadenylation site can vary in a single cell. For example, in Cell #2–10, three different variations of the 3′ end of *β22* were detected. Two of the three transcripts utilized the same polyadenylation signal, but the polyadenylation was added on a different nucleotide, while the other transcript utilized a different polyadenylation signal (Figure 5). This finding shows that even within a single cell the polyadenylation site can be variable, and suggests that the polyadenylation of the 3′ ends of *Pcdh-β* transcripts may be tightly or stochastically regulated.

A previous study detected the expression of several members of the *Pcdh-β* genes in mouse and human brain as a single exonic and 3′-polyadenylated form (Vanhalst et al., 2001). Furthermore, several studies have reported the expression of *Pcdh-β* transcripts in the central nervous system including rat brain, rat hippocampus, and mouse retina (Sago et al., 1995; Schippert et al., 2009; McGowan et al., 2011). Our results confirm these findings and expand them to show that some, if not all, of the 22 *Pcdh-β* genes are expressed in the central and peripheral nervous system, including the cerebellum, hippocampus, cerebral cortex, trigeminal ganglion, dorsal root ganglion, and brain stem.

Focusing on the individual *Pcdh-β* genes, our findings of random-like and combinatorial expression of the individual *Pcdh-β* genes extensively broaden the potential for *Pcdh*-driven single-neuron diversity. The random-like scattered expression of individual *Pcdh-α* and *Pcdh-γ* genes has been previously observed in olfactory bulb periglomerular neurons, hippocampal neurons, Purkinje cells, dorsal root ganglia neurons, and choroid plexus epithelial cells (Kohmura et al., 1998; Esumi et al., 2005; Frank et al., 2005; Kaneko et al., 2006; Prasad and Weiner, 2011; Lobas et al., 2012). In this study, we observed that individual *Pcdh-β* genes are expressed with a similar scattered pattern in cerebellar Purkinje cells, hippocampus, cerebral cortex, trigeminal ganglia, dorsal root ganglia, GABAergic interneurons, and cholinergic neurons. This pattern persisted during development, in that the random-like scattered expression of the *Pcdh-β* genes in cerebellar Purkinje cells was observed from P10 to P150. Furthermore, our data clearly showed that the individual *Pcdh-β* genes are regulated monoallelically and combinatorially in cerebellar Purkinje cells, similar to the *Pcdh-α* and *Pcdh-γ* genes.

Our present results suggest that the individual *Pcdh-β* genes are differentially expressed among the various neuron types in the central and peripheral nervous systems from the juvenile to adult stages, and imply that the *Pcdh-β* gene cluster is regulated by similar mechanisms to the *Pcdh-α* and *Pcdh-γ* genes. The similar genomic organization, involving multiple promoters with a conserved sequence element (CGCT), long-range enhancer element, and multiple CTCF-binding sites, supports this hypothesis (Wu et al., 2001; Ribich et al., 2006; Handoko et al., 2011; Yokota et al., 2011). To date, we have not found any rules governing the spatial distribution of the *Pcdh-β* transcripts; however it remains to be elucidated whether or not the pattern is truly random. One intriguing possibility is that the *Pcdh-β* expressional repertoires in individual neurons determine their target status during neural circuit formation; i.e., pre- and post-synaptic neurons might express the same sets of *Pcdh-β* genes. The present finding that *Pcdh-β* genes show a scattered expression pattern on possible pre- and post-synaptic neurons in the hippocampal inhibitory interneuron network (Figures 6D–I and 8B,C,E,F,H,I) supports this idea.

The present study showed that the expression of the *Pcdh-β* gene cluster is monoallelic and combinatorial in single Purkinje cells. This strongly suggests that individual Purkinje cells express a variable repertoire of some, if not all, of the 22 *Pcdh-β* genes. In this study, we investigated six of the 22 *Pcdh-β* genes and showed that at least two types of *Pcdh-β* per allele can be expressed by a single Purkinje cell (see *β9* and *β19* in Cell #1–17, and *β3* and *β9* in Cell #2–22). The variety of *Pcdh-β* species expressed by a single Purkinje cell can be extrapolated from the present results. In the experiments shown in Figures 3 and 4, among the 28 Purkinje cells analyzed, in 18 of them, one or more clear bands representing the PCR product were obtained from all three split tubes (*β10* in Cell #1–1, *β15* in Cell #1–2, *β19* in Cell #1–5, *β9* in Cell #1–7, *β9* and *β19* in Cell #1–10, *β19* in Cell #1–11, *β3* and *β22* in Cell #1–12, *β3* in Cell #1–14, *β9* and *β19* in Cell #1–17, *β15* in Cell #1–22, *β15* in Cell #1–26, *β10* in Cell #1–28, *β9* from biallelic expression in Cell #1–36, *β19* in Cell #1–39). Therefore, 0.64 (18/28) *Pcdh-β* genes were expressed per Purkinje cell. Assuming that there is no distortion of expression frequencies between isoforms, and given that there are 22 genes in the *Pcdh-β* cluster, we can estimate that 2.3 *Pcdh-β* genes (0.64 multiplied by 22/6) should be expressed per individual Purkinje cell. Thus, at least two *Pcdh-β* isoforms would be expressed by any given Purkinje cell. If these assumptions prove correct, the *Pcdh-β* transcripts could, in theory, produce more than 462 (22 × 21) possible combinations (one *Pcdh-β* isoform from each allele) in single Purkinje cells.

The combinatorial co-expression of the mouse *Pcdh* cluster genes, including the *Pcdh-α*, *Pcdh-β*, and *Pcdh-γ* genes, could provide diversity sufficient to represent more than 20 million unique Purkinje cells, among which *Pcdh-α* transcripts could produce 132 (12 × 11) possible combinations (one *Pcdh-α* isoform from each allele), *Pcdh-β* transcripts could produce 462 (22 × 21) possible combinations (one *Pcdh-β* isoform from each allele), and *Pcdh-γ* transcripts could produce 342 (19 × 18) possible combinations (one *Pcdh-γ* isoform from each allele). Interestingly, rats have about 500 thousand cerebellar Purkinje cells, and humans have 14 million (Ito, 1984). Thus, the possible combinations of *Pcdh* cluster gene expressions would be sufficient to distinguish each cell.

The hypothesis that the *Pcdh* cluster genes play roles in providing cell-surface diversity is supported by reports that showed synaptic and surface localization of the Pcdh-α, Pcdh-β, and Pcdh-γ proteins and their formation of multimeric complexes (Kohmura et al., 1998; Wang et al., 2002b; Murata et al., 2004; Junghans et al., 2008; Chen et al., 2009; Fernandez-Monreal et al., 2009; Han et al., 2010; Puller and Haverkamp, 2011). Schreiner and Weiner (2010) showed that seven Pcdh-γ members exhibit isoform-specific homophilic binding, and that heteromultimeric *cis*-tetramers function as a homophilic binding unit. Although whether heteromultimeric *cis*-tetramers include Pcdh-β proteins is unclear, it is an intriguing possibility that heteromultimeric *cis*-tetramers with Pcdh-β proteins extend the homophilic binding specificity and play important roles in neural circuit formation.

This scenario could be supported by the following two findings. First, Pcdh-β proteins associate with Pcdh-γ proteins (Han et al., 2010). Second, the Cys-(X)5-Cys (C-X5-C) motif is important for the formation and cell-surface expression of covalently bound *cis*-tetramers, and the C-X5-C motif in the EC1 domain is completely conserved among all clustered Pcdh proteins in vertebrates (Schreiner and Weiner, 2010; Yagi, 2012). Our group plans to examine the formation of heteromultimeric *cis*-tetramers that include Pcdh-β proteins and their binding activities.

The importance of highly diverse transmembrane molecules in the formation of neuronal circuits has been shown in *Drosophila*, where *Dscam1* molecules are responsible for the correct patterning of neuronal circuits (Hattori et al., 2007, 2008; Schmucker and Chen, 2009). We therefore speculate that the requirement for single-neuron diversity in neural circuit formation is conserved from insects to vertebrates, and that the Pcdh cluster is responsible for this role in the vertebrate nervous system (Zipursky and Sanes, 2010; Yagi, 2012). Future studies aimed at testing this hypothesis could shed light on the fundamental mechanisms underlying vertebrate neural circuit formation.

### Conflict of interest statement

The authors declare that the research was conducted in the absence of any commercial or financial relationships that could be construed as a potential conflict of interest.

## Figures and Tables

**Figure S1:** **Partial sequence, of a region close to the 3-polyadenylated site, of some of the *Pcdh-β* genes analyzed in this study**. We found 27 polymorphisms (indicated by a yellow background) that enabled us to distinguish whether the amplified product was from the B6 or JF1 chromosome. The putative polyadenylation signal sequences are shown in red-underlined letters.

**Table S1:** **List of primers**.

**Table S2:** **The possible poly adenylation signals of some of the *Pcdh-β* transcripts**.
